# An observation of the peer-assisted learning (PAL) method in the clinical teaching of vertigo/dizziness-related diseases for standardized residency training (SRT) students in China: a randomized, controlled, multicenter study

**DOI:** 10.1186/s12909-021-02969-1

**Published:** 2021-10-14

**Authors:** Rui Xu, Chunmei Duan, Qian He, Zhaoyou Meng, Gong Wang, Shu Liu, Meng Guo, Xiaoyan Chen, Yue Wang, Wei Duan, Qin Zhang, Qingwu Yang, Xiaojun Liang, Yang Bai

**Affiliations:** 1 Department of Neurology, The Second Affiliated Hospital, Army Medical University, Chongqing, 400037 China; 2 Department of Neurology, Chongqing Traditional Chinese Medicine Hospital, Chongqing, 400021 China; 3 Department of Otolaryngology, The Second Affiliated Hospital, Army Medical University, Chongqing, 400038 China; 4 Department of Otolaryngology, The First Affiliated Hospital, Army Medical University, Chongqing, 400038 China

**Keywords:** Peer group, Internship and residency, Vertigo, Dizziness, Medical education

## Abstract

**Background:**

Vertigo and dizziness (VD) are among the most frequently seen symptoms in clinics and are important for medical students, especially for those in Chinese standardized residency training (SRT). The aim of our study was to examine the PAL method’s feasibility in the clinical teaching of VD-related diseases for SRT students in China.

**Methods:**

This is a randomized, controlled, multicenter study. A total of 228 residents were invited to participate in this study, of which 198 completed the program. The students were randomized into two groups, and VD-related diseases were taught using lecture-based learning (control group) or peer-assisted learning (PAL). An examination paper and a rating scale were used to evaluate students’ performance in the mastery of VD-related theoretical knowledge and clinical skills, meanwhile students’ perceptions, satisfaction, and risk of burnout were also analyzed using a questionnaire. Independent-samples *t*-test and chi-square analysis were performed to evaluate statistical significance for continuous variables and categorical variables, respectively, using SPSS 18.0 software.

**Results:**

The PAL group performed better in mastering theoretical knowledge and clinical skills than the control group. And more students believed that PAL could help improve their personal qualities such as teamwork skills. However, more students reported that PAL increased the risk of burnout.

**Conclusions:**

PAL was a suitable and effective method in the clinical teaching of some specialized diseases, especially it was recommended for students who had gained initial knowledge and skills, such as Chinese SRT students. However, we should draw attention to the increased risk of burnout if PAL is intended to be widely used in clinical teaching.

**Trial registration:**

ISRCTN registry, ISRCTN53773239, 05/07/2021, retrospectively registered.

**Supplementary Information:**

The online version contains supplementary material available at 10.1186/s12909-021-02969-1.

## Introduction

A new standardized residency training (SRT) program for medical trainees was initiated in China in recent years. This new program aimed to create more standardization in physicians’ training and improve the clinical skills of students who previously completed basic theoretical courses and accumulated internship experience in a medical college [1, 2]. Therefore, it is important for residents to receive systematic training to gain overall improvement in professional competency, including theoretical knowledge and clinical skills [2, 3]. This is especially true for some specialized diseases, such as those related to vertigo/dizziness (VD). And in this way, SRT helps to enhance the quality of medical services offered in China.

VD are among the most frequently reported symptoms in emergency room (ER) consultations. However, distinguishing between serious and benign disorders caused by VD remains challenging in emergency consultations, even for experienced doctors. Additionally, misdiagnosis may be fatal for patients, e.g., serious disorders caused by a posterior stroke [4]. Therefore, the challenges of making a fast and correct diagnosis remind us of the importance of the clinical teaching of VD-related diseases.

As VD may be caused by various diseases presenting diverse symptoms and different pathologies, knowledge of neurology, otolaryngology, ophthalmology and psychology is required; hence, difficulties exist in VD-related diseases’ clinical teaching. In traditional teacher-centered education, VD-related diseases have been taught using lecture-based learning (LBL), which often lacks clinical relevance and may predispose learners to information overload [5]. Furthermore, this teaching method is considered to induce passivity and compliance, focusing on a one-way transfer of knowledge [6]. For Chinese SRT students, our experience also indicated that traditional LBL method could not achieve the desired outcomes in the clinical teaching of VD-related diseases. It is, therefore, necessary to try another teaching method to obtain better teaching results.

Peer-assisted learning (PAL), which is defined as “People of similar social groupings who are not professional teachers helping each other to learn and learning themselves by teaching” [7], is an established teaching and learning method used in medical education worldwide [8] that implements peer teaching programs by using students as teachers [9, 10]. PAL has been widely used in medical education and many benefits have been reported by previous studies [11–18]. A conceptual framework suggests that five processes contribute to the effectiveness of PAL, including “organization and engagement, cognitive conflict, scaffolding and error management, communication, and affect”. These processes promote both the “tutor” and the learner to learn in the PAL teaching, and has both cognitive and affective outcomes. Finally, it makes students more aware of PAL method and feeds back to the original five processes in a virtuous cycle, to make PAL more effective [12, 19]. Meanwhile, it was also reported that the secure and collaborative environment in the PAL session also promoted learning [20]. In this way, PAL is mutually beneficial for student tutors and student learners [21–23]. Additionally, unlike traditional LBL, it encourages an active and open exchange of knowledge that improves clinical teaching quality [5, 10].

As SRT students in China who have already finished basic theoretical courses and accumulated internship experience, PAL may be suitable for them especially in the clinical teaching of some complicated and specialized diseases, such as VD-related disease. And to the best of our knowledge, PAL was used and compared with traditional teaching methods in other areas of medical education like osteology, dental medicine and nursing [12, 24, 25], there is a lack of research assessing whether PAL can be used in the SRT program or the clinical teaching of VD-related diseases. Therefore, this study aimed to compare teaching results using the PAL method with the LBL method and examine the PAL method’s feasibility in the clinical teaching of VD-related diseases for SRT students in China.

## Methods

### Design

This is a randomized, controlled, multicenter study conducted in three hospitals in Chongqing, China, including four SRT training sites certified by the Chongqing Municipal Health Commission.

### Participants and setting

A total of 228 residents studying at these four SRT training sites between January 2017 and December 2020 were invited to participate in the study (30 residents were ultimately excluded). The inclusion criteria were as follows: active residents employed in four training sites who were willing to participate in the training program and assessment procedure. The following exclusion criteria were used: residents who had previously participated in similar curriculum and residents who completed less than 50% of the training program.

The sample size and power analysis were calculated using “Compare 2 Means: 2-Sample, 2-Sided Equality” tool in the “Power and Sample Size” website (http://powerandsamplesize.com/Calculators/) according to our preliminary experiment results. Briefly, this calculator uses the following formulas to compute sample size and power, respectively:

$${n}_A=\kappa {n}_B\kern0.5em \mathrm{and}\kern0.5em {n}_B=\left(1+\frac{1}{\kappa}\right){\left(\sigma \frac{z_{1-\alpha /2}+{z}_{1-\beta }}{\mu_A-{\mu}_B}\right)}^2$$

$$1-\beta =\Phi \left(z-{z}_{1-\alpha /2}\right)+\Phi \left(-z-{z}_{1-\alpha /2}\right),\kern0.5em z=\frac{\mu_A-{\mu}_B}{\sigma \sqrt{\frac{1}{n_A}+\frac{1}{n_B}}}$$

Where:

- κ = n_A_/n_B_ is the matching ratio
- σ is standard deviation
- Φ is the standard Normal distribution function
- Φ^− 1^ is the standard Normal quantile function
- α is Type I error
- β is Type II error, meaning 1 − β is power

Seven attending doctors and 6 senior doctors were invited as directors. All of the residents had completed basic theoretical courses and clinical clerkships in a medical college.

### Ethical consideration

The present study was approved by the Ethics Committee of Xinqiao Hospital, Army Medical University. Participants, who had been previously informed about the project, voluntarily participated this project and agreed with the randomization. Meanwhile participant could quit the project at any time during the research. Written informed consent was obtained from all participants. Meanwhile all the researchers signed a confidentiality agreement regarding the participants’ data.

### Intervention and control groups

The students and directors were randomized into two groups: the control (LBL) group and the PAL group. There were 99 residents in each group. The program was completed in 2 weeks.

In the *control group*, i.e., the PBL group, the program was teacher-centered, and the lecture-based teaching method was used for theoretical knowledge teaching. The directors taught epidemiology, definitions, classifications, pathophysiology, diagnosis and differential diagnosis, and treatment of VD-related diseases. For clinical practice teaching, different typical cases of VD-related patients were chosen and taught by the directors.

In the *PAL group*, the 99 students were randomized into 33 subgroups (3 students in one subgroup). One student in the subgroup was selected as the tutor by other members. All of the selected student tutors received routine tutor training that comprised three individual 3-h sessions led by directors, as previously reported [10]. After that, the students learned knowledge of VD-related diseases, and then the theoretical knowledge was taught by the student tutor, followed by a group discussion. For clinical practice, the same VD-related patient cases in the control group were chosen. The students performed a medical history inquiry and physical examination by themselves, followed by a group discussion under a student tutor’s organization. Finally, each group worked as a team to come up with a diagnosis and treatment plan. The entire process was supervised and commented on by one director.

### Instruments and data collection

#### Examination performance

Examination performance was evaluated before and after teaching.

Mastery of theoretical knowledge was assessed through a written examination paper consisting of 40 single best answer questions that contained four parts (“*Concept and classification of VD-related diseases*,” “*Symptoms of different VD-related diseases*,” “*Signs of different VD-related diseases*”, and “*Treatment of different VD-related diseases*”, with 2 points awarded for each question and 10 questions for each part) and 2 open-ended questions (“*Diagnosis and differential diagnosis*”, with 10 points for each question). All the questions were chosen from our examination database of neurology. The examination questions in the database were classified into different difficulty levels, and questions were randomly chosen from different levels and constituted a final examination paper for all of the participants enrolled in this research. Details of the paper were shown in supplementary materials.

Clinical skills were also examined by analyzing the case of a standardized patient. The evaluation was performed in five parts: “*Medical history inquiry*,” “*Physical examination*,” “*Diagnosis and treatment plan*,” “*Communication skills and care for patients,*” and “*Overall evaluation*” (20 points for each part). The rating scale used for clinical skills evaluation of VD-related diseases was given in supplementary materials.

Examination was performed within 1 week before and after teaching. The examination performance was evaluated by the teaching secretaries (they are also clinical doctors) blinded to the group assignments.

#### Questionnaire

##### Student perceptions and satisfaction

The students completed a researcher-made questionnaire (3-point scale with 1 = improved and 3 = not improved) asking about their perceptions of the teaching process from 7 aspects. This questionnaire included items about “*Study activeness*,” “*Mastery of theoretical knowledge*,” “*Efficiency of learning*,” “*Application of clinical knowledge*,” “*Communication skills*,” “*Self-study ability*,” and “*Self-confidence and teamwork skills*.” The items and scopes of the questionnaire were chosen according to previous report [1, 26] and our own teaching experience.

Additionally, we investigated satisfaction and the risk of burnout using different teaching methods with a researcher-made questionnaire (3-point scale with 1 = agree and 3 = disagree).

Questionnaire was completed within 1 week after teaching.

##### Validity and reliability

The responses on the scale in the questionnaire were collected, and the validity and reliability were analyzed using SPSS 18.0 software.

### Statistical methods

The basic characteristics of each cohort are presented as the mean ± SD. For continuous variables (such as score), an independent-samples *t*-test was used to evaluate statistical significance between groups, while a paired-samples *t*-test was used to analyze the paired data (pretest and posttest). For categorical variables (such as sex), chi-square analysis was used to evaluate statistical significance. The statistical method used for variable with three categories was also chi-square analysis in Descriptive Statistics → Crosstabs. If any cells had expected count less than 5, Fisher’s Exact Test would be applied. *P*-values less than 0.05 were considered significant. Analyses were performed with SPSS 18.0 software.

## Results

### Characteristics of residents

**Fig. 1:**
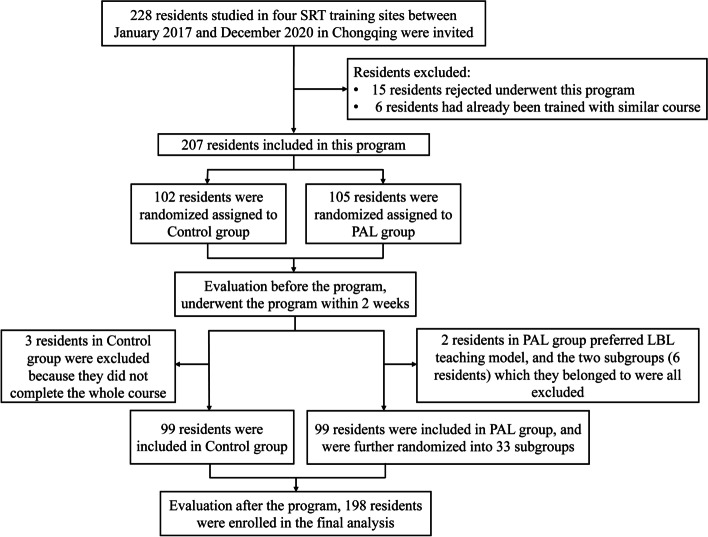
Flow chart of the study. Abbreviations: SRT: standardized residency training; PAL: peer-assisted learning; LBL: lecture-based learning

A power analysis was carried out to calculate the sample size (power = 0.9, *n* = 110; power = 0.8, *n* = 83). And A total of 228 residents were invited to participate in this study, of which 21 residents were excluded and 9 residents were dropped out during research process (details were specified in Fig. 1). Process of randomization and definitive participation of students is shown in Fig. 1. Residents were from different specialties at four separate training sites. The demographic and work characteristics of the residents are summarized in Table 1. The Control group and PAL group’s mean ages were similar (24.65 years vs. 24.47 years). Other important basic characteristics (such as work experience, degree of education, and year in SRT) that might lead to the risk of bias were all assessed. The results indicated that the two groups’ basic characteristics were similar without significant differences (*p* > 0.05).

**Table 1:** Demographic and work characteristics of residents and statistical analysis

Variables	Control (***n*** = 99) No. (%)	PAL (***n*** = 99) No. (%)	P*
**Age** (years, mean ± SD)	24.65 ± 2.06	24.47 ± 2.14	0.70
**Sex (M/F)**			0.39
Male	45 (45.45)	51 (51.51)	
Female	54 (54.55)	48 (48.48)	
**Work experience before SRT**			0.64
Yes	11 (11.11)	9 (9.09)	
None	88 (88.89)	90 (90.91)	
**Education**			0.49
Bachelor’s degree	60 (60.61)	68 (68.69)	
Master’s degree	32 (32.32)	25 (25.25)	
Doctoral degree	7 (7.07)	6 (6.06)	
**Year in SRT**			0.57
1st year	32 (32.32)	39 (39.39)	
2nd year	35 (35.35)	30 (30.30)	
3rd year	32 (32.32)	30 (30.30)	
**Recognition on necessity of SRT**			0.74
Unnecessary	10 (10.10)	9 (9.09)	
Neutral	65 (65.66)	70 (70.71)	
Necessary	24 (24.24)	20 (20.20)	

^*^Independent-samples *t*-test for continuous variables (age); Chi-square analysis for categorical variables (sex)

### Examination performance

Regarding mastery of theoretical knowledge, it was found that before teaching, no significant differences in the scores were found between the Control group and the PAL group (except for “*Diagnosis and differential diagnosis*,” Table 2). After teaching, test scores were significantly higher in both groups compared with before teaching (statistical data not shown in the Table 2). However, students in the PAL group performed significantly better in examining theoretical knowledge than those in the Control group. The PAL group had higher scores than the Control group in the following five parts: “*Concept and classification of VD-related diseases*” (Table 2: Control vs. PAL, 13.01 ± 3.27 vs. 15.23 ± 2.84; **p < 0.01), “*Symptoms of different VD-related diseases*” (Table 2: Control vs. PAL, 11.35 ± 2.57 vs. 12.53 ± 2.13; **p < 0.01), “*Signs of different VD-related diseases*” (Table 2: Control vs. PAL, 11.49 ± 2.67 vs. 15.58 ± 2.68; **p < 0.01), “*Diagnosis and differential diagnosis*” (Table 2: Control vs. PAL, 12.59 ± 2.06 vs. 13.85 ± 1.97; **p < 0.01), and “*Treatment of different VD-related diseases*” (Table 2: Control vs. PAL, 11.17 ± 2.63 vs. 13.43 ± 2.68; **p < 0.01). The average total score of theoretical knowledge for the PAL group was 70.62, which was also significantly higher than that of the Control group (average score: 59.62, **p < 0.01; Table 2).

**Table 2:** Comparison of scores (theoretical knowledge) between two groups before and after teaching

Items	Pretest				Posttest			
	Control	PAL	***t***	***P***	Control	PAL	***t***	***P***
*Concept and classification of VD-related diseases*	8.85 ± 3.00	8.24 ± 2.80	1.47	0.14	13.01 ± 3.27	15.23 ± 2.84	−5.10	< 0.01
*Symptoms of different VD-related diseases*	8.08 ± 3.17	8.00 ± 2.41	0.20	0.84	11.35 ± 2.57	12.53 ± 2.13	−3.49	< 0.01
*Signs of different VD-related diseases*	7.96 ± 1.67	7.68 ± 2.09	1.05	0.29	11.49 ± 2.67	15.58 ± 2.68	−10.72	< 0.01
*Treatment of different VD-related diseases*	8.44 ± 2.28	8.34 ± 2.10	0.32	0.75	11.17 ± 2.63	13.43 ± 2.68	−5.99	< 0.01
*Diagnosis and differential diagnosis*	9.32 ± 1.92	8.66 ± 1.73	2.56	0.01	12.59 ± 2.06	13.85 ± 1.97	−4.41	< 0.01
*Total score*	42.66 ± 6.68	40.92 ± 5.77	1.96	0.06	59.62 ± 6.78	70.62 ± 6.03	−12.06	< 0.01

Improvements in clinical skills after teaching were also investigated. As shown in Table 3, the exam results revealed that both groups’ scores increased after teaching. More importantly, it was found that the average scores for “*Medical history inquiry*,” “*Physical examination*,” “*Diagnosis and treatment plan*,” “*Communication skills and care for patients,*” and “*Overall evaluation*” in the PAL group were 15.08, 15.13, 15.53, 13.81 and 14.76 points, respectively, which were higher than those in the Control group (13.11, 12.69, 11.27, 12.19 and 13.09, respectively, **p < 0.01; Table 3). The PAL group’s average total score also increased significantly compared with that of the Control group (74.30 vs. 62.35, **p < 0.01, Table 3).

**Table 3:** Comparison of scores (clinical skills) between two groups before and after teaching

Items	Pretest				Posttest			
	Control	PAL	***t***	***P***	Control	PAL	***t***	***P***
*Medical history inquiry*	8.93 ± 2.35	7.44 ± 2.00	4.79	< 0.01	13.11 ± 2.58	15.08 ± 1.54	−6.51	< 0.01
*Physical examination*	8.11 ± 2.01	8.00 ± 2.58	0.34	0.74	12.69 ± 2.32	15.13 ± 2.01	−7.92	< 0.01
*Diagnosis and treatment plan*	6.86 ± 2.21	8.51 ± 1.53	−6.09	< 0.01	11.27 ± 2.48	15.53 ± 1.71	−14.04	< 0.01
*Communication skills and care for patients*	7.27 ± 2.69	8.37 ± 1.93	−3.31	< 0.01	12.19 ± 2.24	13.81 ± 2.28	−5.04	< 0.01
*Overall evaluation*	10.25 ± 1.45	8.43 ± 1.14	9.81	< 0.01	13.09 ± 1.33	14.76 ± 1.18	−9.32	< 0.01
*Total score*	41.42 ± 6.49	40.76 ± 4.64	0.83	0.41	62.35 ± 6.53	74.30 ± 5.25	−14.19	< 0.01

After the study was finished, power was also calculated and the power was 0.99 for the primary outcome, i.e., examination performance.

### Questionnaire

#### Validity and reliability

The validity and reliability were analyzed, and Kaiser-Meyer-Olkin (KMO) for Control group and PAL group were 0.907 and 0.888, respectively. Meanwhile Cronbach’s Alpha for Control group and PAL group were 0.974 and 0.968, respectively.

#### Student perceptions and satisfaction

When the teaching program finished, a list of potential concerns focusing on different attitudes and effects was ranked through a questionnaire, which was evaluated by the students. It revealed that more students believed that PAL could help improve their “*Study activeness*,” “*Mastery of theoretical knowledge*,” “*Efficiency of learning*,” “*Application of clinical knowledge*,” “*Self-study ability*,” and “*Self-confidence and teamwork skills*” (Table 4). More students in the PAL group (55.56%) than that in the Control group (44.44%) were satisfied and recommended how they were taught. However, it should also be noted that 62.63% of students reported that PAL increased the risk of burnout, which was more than that of students in the Control group (19.19%, **p < 0.01, Table 5).

**Table 4:** Residents’ perceptions of the teaching process [n (%)]

Items	Control			PAL			***χ***^**2**^	***P***
	Improved	Neutral	Not Improved	Improved	Neutral	Not Improved		
*Study activeness*	45 (45.45%)	45 (45.45%)	9 (9.09%)	60 (60.61%)	37 (37.37%)	2 (2.02%)	7.38	0.03
*Mastery of theoretical knowledge*	30 (30.30%)	49 (49.49%)	20 (20.20%)	62 (62.63%)	36 (36.36%)	1 (1.01%)	30.31	< 0.01
*Efficiency of learning*	30 (30.30%)	52 (52.53%)	17 (17.17%)	63 (63.64%)	36 (36.36%)	0 (0%)	31.62	< 0.01
*Application of knowledge in the clinical*	32 (32.32%)	51 (51.51%)	16 (16.16%)	57 (57.58%)	40 (40.40%)	2 (2.02%)	19.24	< 0.01
*Communication skills*	23 (23.23%)	69 (69.70%)	7 (7.07%)	25 (25.25%)	70 (70.71%)	4 (4.04%)	0.91	0.64
*Self-study ability*	25 (25.25%)	49 (49.49%)	25 (25.25%)	40 (40.40%)	59 (59.60%)	0 (0%)	29.39	< 0.01
*Self-confidence & Teamwork skills*	25 (25.25%)	59 (59.60%)	15 (15.15%)	50 (50.51%)	49 (49.49%)	0 (0%)	24.26	< 0.01

**Table 5:** Risk for burnout and satisfaction of different methods [n (%)]

**Items**	**Control**			**PAL**			***χ***^**2**^	***P***
	**Agree**	**Neutral**	**Disagree**	**Agree**	**Neutral**	**Disagree**		
*Increased risk for burnout*	19 (19.19%)	65 (65.66%)	15 (15.15%)	62 (62.63%)	37 (37.37%)	0 (0%)	45.51	< 0.01
	**Recommended**	**Neutral**	**Not Recommended**	**Recommended**	**Neutral**	**Not Recommended**	***χ***^**2**^	***P***
*Satisfaction and recommendation of PAL method*	44 (44.44%)	54 (54.55%)	1 (1.01%)	55 (55.56%)	43 (43.43%)	1 (1.01%)	9.34	< 0.01

## Discussion

VD-related diseases are traditionally taught using the LBL method, which is widely used in medical education. The LBL method has recently been considered a one-way transfer of knowledge that may predispose learners to information overload and promote a passive learning culture [5, 6]. Our previous experience also indicated that clinical teaching of VD-related diseases using the traditional LBL method did not achieve the desired outcomes, and more variety in teaching approaches was needed.

In this study we implemented PAL in the clinical teaching of VD-related diseases for SRT students in China. Compared with the traditional LBL method, the PAL method promoted mastery of VD-related knowledge and skills and improved students’ personal qualities. PAL activities encompass “*People from similar social groupings who are not professional teachers helping each other to learn and learning themselves by teaching*” [27]. As mentioned above, students participating in SRT have already finished basic theoretical courses and accumulated internship experience; thus, initial knowledge and skills are gained, and it is suitable for the implementation of PAL in the SRT program. Previous similar articles reported application of PAL for undergraduate students’ education in Australia, India, Pakistan, etc. And in these studies PAL was used in the area of radiography, osteology, and dental education, respectively [12, 24, 28]. However, to the best of our knowledge, few studies assess how the PAL method works in SRT in China, as well as clinical teaching of VD-related disease.

In our study, VD-related disease is considered complicated and difficult to learn for residents. We tried to teach students in this new way because student tutors and student learners had a similar knowledge base and learning experiences, i.e., they “were on the same wavelength” [10]. Therefore, student tutors could determine which part of VD-related knowledge was challenging to learn for student learners. With the help of directors, student tutors could explain key points in a way that was easier for student learners to understand [23]. Additionally, student tutors and student learners have similar social roles, which encouraged an open exchange of ideas and concerns. In this way, student tutors and student learners would have sufficient discussion opportunities during the teaching process and learn from each other. Our study confirmed that the PAL method finally promoted mastery of VD-related knowledge and skills. In line with our study, PAL group showing higher mean scores in osteology teaching, compared with traditional didactic methods [12]. Not only in the area of medicine, but also PAL was demonstrated a useful tool in the other areas of health sciences. For example, Nasenien et al. demonstrated that PAL was a useful tool for teaching basic abdominal ultrasound [29]; George reported that PAL was a beneficial teaching strategy for nursing students [25]. However, inconsistent with our results, Ambreen et al. reported that in dental education, test scores, academic performance and overall learning in PAL sessions was not higher than control group. But they still recommended that PAL could be utilized as an important supplement to synchronous teaching tele-presence, especially during current pandemic situation [24].

When a new method is implemented in clinical teaching, results on student satisfaction and perceptions should be noted, as student satisfaction and engagement lead to achieving program outcomes [30–32]. In our study, student perceptions and satisfaction were investigated using a self-evaluated questionnaire. According to students’ perceptions, mastery of professional skills was promoted, and qualities such as self-study, self-confidence, and teamwork skills were improved. In line with our findings, Wawrzynski et al. reported that peer education improved students’ public speaking skills, organizational abilities, and individual self-confidence [33]. However, a potential concern raised is the increased risk of burnout. Burnout is a common experience for medical residents that leads to many challenges, including mental health issues such as suicidal tendencies and physical symptoms such as fatigue and headaches [34–38]. In our study, although most of the students had accepted the PAL method, it should be noted that PAL increased the risk of burnout. This phenomenon can be explained by long hours of self-study after class, concurrent with preparation for teaching and class discussion, which might also increase studying-related pressure. Therefore, we should draw attention to the increased risk of burnout if PAL is intended to be widely used in clinical teaching. Same concern was also raised by other researchers, Hundertmark et al. comprehensively quantified tutors’ stress and described frequent stressors in the PAL [39], and Matthew et al. proposed that support and feedback developed competence and confidence and reduced stress and anxiety [40].

Although PAL has been used worldwide, the present study is, to our knowledge, the first randomized, controlled, multicenter study to explore its implementation in SRT programs in China and the first study to explore its potential application in the clinical teaching of VD-related diseases. However, our study has some limitations. For instance, student tutors were chosen by students rather than arranged randomly, therefore, “high achievers” with high confidence who were often selected as tutors while students with low confidence might have been ignored. However, those who did not volunteer are probably the ones who needed the exercise most. Therefore, improving the personal skills of students with low confidence by having them work as student tutors could not be investigated in this study.

## Conclusions

The paper describes implementing PAL in the clinical teaching of VD-related diseases for SRT students in China. Compared with the traditional LBL method, the PAL method promoted mastery of VD-related knowledge and skills and improved students’ personal qualities, such as self-confidence, communication, self-study, and teamwork skills. Most of the students were satisfied and recommended the PAL method. However, our study also reminded us to draw attention to the increased risk of burnout when teaching using the PAL method. Overall, our findings suggested that PAL was a suitable and effective method in the clinical teaching of some specialized diseases, especially for students who had already gained initial knowledge and skills, such as SRT students in China.

## Supplementary Information

**Additional file 1: Table S1.** The paper used for theoretical knowledge examination of VD-related diseases. **Table S2.** The rating scale used for clinical skills evaluation of VD-related diseases.

## Data Availability

The data analyzed under the current study are available from the corresponding author on reasonable request (Bai Yang, Email: bessie1011@163.com).

## Abbreviations

SRT: Standardized residency training
VD: Vertigo and dizziness
LBL: Lecture-based learning
PAL: Peer-assisted learning
