# Supplementary material for: An observation of the peer-assisted learning (PAL) method in the clinical teaching of vertigo/dizziness-related diseases for standardized residency training (SRT) students in China: a randomized, controlled, multicenter study
Source: BMC Med Educ. 2021 Oct 14;21:532. doi: 10.1186/s12909-021-02969-1 (PMC8518317; doi:10.1186/s12909-021-02969-1)
Supplement: Supplementary file 1 — Additional file 1: Table S1. The paper used for theoretical knowledge examination of VD-related diseases. Table S2. The rating scale used for clinical skills evaluation of VD-related diseases. [file 12909_2021_2969_MOESM1_ESM.docx]

Table S1 The paper used for theoretical knowledge examination of VD-related diseases

| **Themes** | **Examples** | |
| --- | --- | --- |
|  | **Question** | **Single best answer** |
| *Concept and classification of VD-related diseases* | Acute vertigo can be subdivided into two main groups: (1) ＿ and (2) provoked vertigo. | Spontaneous vertigo |
|  | ＿ is characterized by brief attacks of rotatory vertigo with torsional positioning nystagmus, which are elicited by changes in the head position relative to gravity. | BPPV |
| *Symptoms of different VD-related diseases* | In BPPV, the vertigo appears with short latency, lasts for ＿ and is characterized by an increase followed by a decrease in its intensity | less than a minute |
|  | Transient VD with hearing loss or other ear-related symptoms and a history of at least one other similar attack, it most likely to be ＿. | Menière’s disease |
| *Signs of different VD-related diseases* | Vertigo and dizziness caused by posterior circulation stroke, neurological signs could usually be found (e.g., double vision, dysarthrophonia or ＿). | hemiparesis |
|  | For Posterior-canal-type of BPPV, torsional nystagmus, in which the upper pole of the eye rotates toward the ＿ ear, is induced by the ＿maneuver where the patient is brought from the upright to supine position with the head turned 45° to the affected ear. | Affected,  Dix–Hallpike |
| *Treatment of different VD-related diseases*, | ＿ is used to decrease neurovegetative symptoms and general patient distress. | Methoclopramide |
|  | Diazepam can be used to decrease ＿ in the acute phase of vertigo and dizziness. | internuclear inhibition |
|  |  |  |
| **Themes** | **Examples** | |
|  | **Question** | **Open-ended answer** |
| *Diagnosis and differential diagnosis* | What is the criteria for diagnosis of definite Menière’s disease? | (1) Two or more spontaneous episodes of vertigo, each lasting 20 minutes to 12 hours.  (2) Audiometrically documented low- to medium-frequency sensorineural hearing loss in one ear, defining the affected ear on at least one occasion before, during or after one of the episodes of vertigo.  (3) Fluctuating aural symptoms (hearing, tinnitus or fullness) in the affected ear.  (4) Not better accounted for by another vestibular  diagnosis. |
|  | Please explain the definition and diagnostic criteria of orthostatic vertigo. | Dizziness related to orthostatic challenges (getting up from a lying or sitting position) with pathological orthostasis test (drop of systolic blood pressure> 20 mmHg within 3 min after changing into an upright position). |

Table S2 The rating scale used for clinical skills evaluation of VD-related diseases

| **Themes** | **Details** | **Scores** |
| --- | --- | --- |
| *Medical history inquiry* | Self-introduction and patient’s information verification | 2 |
|  | Chief complaint, main symptoms | 6 |
|  | Accompanying symptoms | 4 |
|  | Negative symptoms | 3 |
|  | History of hospital visiting, diagnosis and treatment | 2 |
|  | Past medical history, personal history, obsterical history and family history | 3 |
| *Physical examination* | Vital signs | 3 |
|  | Neurological examination including nystagmus, finger-nose test, signs of hemiparesis, pyramidal sign, etc. | 10 |
|  | Examination of vestibular function like Dix–Hallpike maneuver | 4 |
|  | General physical examination | 3 |
| *Diagnosis and treatment plan* | Diagnosis | 8 |
|  | Differential diagnosis | 4 |
|  | Treatment plan, including further examination plan, pharmacotherapy and rehabilitation | 8 |
| *Communication skills and* *care for patients* | Communication skills | 10 |
|  | Care for patients | 10 |
| *Overall evaluation* | Overall evaluation of the student’s performance in the whole process, including proficiency, time control and right order, etc.. | 20 |
